# Towards Tumor Targeting via Invasive Assay Using *Magnetospirillum magneticum*

**DOI:** 10.3389/fmicb.2021.697132

**Published:** 2021-07-22

**Authors:** Marvin Xavierselvan, Heena R. Divecha, Mamta Hajra, Sushila Silwal, Isaac Macwan

**Affiliations:** ^1^Department of Biomedical Engineering, Tufts University, Medford, MA, United States; ^2^Department of Biomedical Engineering, University of Bridgeport, Bridgeport, CT, United States; ^3^Department of Electrical and Biomedical Engineering, Fairfield University, Fairfield, CT, United States

**Keywords:** AMB-1, tumor targeting, VMD simulation, magnetotaxis, bacterial invasion

## Abstract

*Magnetospirillum magneticum* (AMB-1) are a species of magnetotactic bacteria (MTB) that are capable of orienting along the earth’s magnetic field lines through their organelles called magnetosomes. Many studies have shown that certain engineered bacteria can infect the tumor cells, resulting in a controlled death of a tumor. This work deals with a technique utilizing AMB-1 along a predefined path through magnetotaxis, which can pave a way for selective doping as well as isolation of the tumor cells from a group of healthy cells through a magnetic invasive assay. For such a control, a tiny mesh of vertical electrical coils each having a diameter of ∼3 mm is fabricated, which establishes the path for the bacteria to move along the magnetic field lines. The molecular dynamics (MD) simulations at the interface of the bacterial cell surface proteins (MSP-1 and flagellin) and Chinese hamster ovary (CHO) cell surface containing cytoplasmic and extracellular proteins (BSG, B2M, SDC1, AIMP1, and FOS) are shown to establish an association between the AMB-1 and the host CHO cells. It is found that the CHO protein structure is compromised, which disables the activation of its defense function, allowing the bacteria to interact and survive. The experimental demonstration involves the CHO cells’ interaction with the AMB-1 and isolation of selected CHO cells. It is found that AMB-1-integrated CHO cells successfully moved along the magnetic field lines generated by the coils. Statistical analysis performed for the assay showed that AMB-1 cells were found to be viable after co-incubating with CHO cells, and the number of viable cells post co-incubation over a period of 24 h showed a slight decrease in both cell population. Overall, 51% of AMB-1 cells and 67% of CHO cells were found viable 24 h post co-incubation. Scanning electron microscopy (SEM) along with energy-dispersive X-ray spectroscopy (EDAX) analysis revealed AMB-1/CHO cell morphology, the potential interaction between them, and the presence of magnetosomes with trace amounts of iron in the AMB-1-interacted CHO cells, confirming the successful AMB-1 integration.

## Introduction

Cancer is a result of uncontrolled cell division and proliferation of cells and is the second leading cause of death in the world (Hanahan and Weinberg, 2011; Siegel et al., 2020). While various therapies have been developed for the treatment, the mainstream therapies are surgery, radiation therapy, chemotherapy, and immunotherapy. Surgeries are only efficient for solid localized tumor and does not possess the spatial control in case of metastasized tumors or leukemia. Furthermore, surgeries cannot eliminate all cancer cells leading to tumor recurrence at later stages. Radiation therapy works by causing irreversible damage to the cell DNA through the creation of reactive oxygen species resulting in disrupted cellular functions and thus damaging their ability to proliferate leading to cell death. The availability of oxygen in the vicinity of the tumor plays an important role as per the studies, where it is shown that the radiation damage caused in the absence of oxygen results in cellular repair and continuous proliferation (Gray et al., 1953; Rockwell et al., 2009). As most of the solid tumor cores are hypoxic, the radiation damages are reversible, leading to the development of radio-resistance (Wouters et al., 2007; Graham and Unger, 2018). Chemotherapy, a non-invasive therapy, utilizes various anti-cancerous drugs for the treatment of cancer throughout the body including the metastasized tumors. The drawbacks of this therapy are the side effects to the normal healthy cells, low efficacy due to small concentration of drugs reaching the core of tumor because of the disrupted vascular network, and the resistance developed by cancer cells against the drugs either intrinsically or owing to the frequent usage or due to hypoxic tumor microenvironment (TME) (Teicher et al., 1981; Wouters et al., 2007; Li et al., 2008; Lippert et al., 2008). Immunotherapy, a fourth pillar in treating cancer, involves the activation of the host immune system against tumors and enhances the killing of tumor cells by blocking various immune checkpoints that the cancer cells use to evade the immune system (Topalian et al., 2012). It is known that the TME that surrounds the tumor contains extracellular matrix, which is composed of a network of proteins, cytokines, and cancer-associated fibroblasts that influences the response to therapy and aids in the proliferation of the cancer cells (Fares et al., 2019). Unfortunately, TME also contains certain immune cells that suppress the response from the immune system (Fridman et al., 2012; Gajewski et al., 2013).

Targeting tumor cells by selective isolation and treatment is a promising strategy, where nanoparticles acting as pH-sensitive or oxygen-sensitive probes carrying drugs to the TME are increasingly being investigated (Gao et al., 2010; Lv et al., 2016; Wu et al., 2018; Wang et al., 2019; Kumari et al., 2020). One way to improve the delivery efficacy is by using receptor-targeted nanoparticles, which are known to selectively deliver the payload to the cancer cells (Zhang et al., 2012; Oh et al., 2018). These targeted nanoparticles enter cells *via* receptor-mediated endocytosis and thereby regular cell internalization is bare minimum. Various studies have been reported for cancer-specific delivery of drugs using targeted nanoparticles and liposomes (Zhang et al., 2012; Shi et al., 2017; Oh et al., 2018). Although selectivity of tumor cells resulting in a lesser toxicity to healthy cells and improved circulation time are achieved with this method, biocompatibility, instability of the nanoparticles, poor drug loading, rapid leakage, and failure to reach the core of TME limit the therapeutic efficacy of these nanoparticle-based drug delivery approaches (Lungu et al., 2019; Navya et al., 2019). Resorting to biological materials can overcome these issues, and several studies have shown the possibility of exploiting biological agents such as cell membrane vesicles, bacteria, and virus for drug delivery (Hosseinidoust et al., 2016; Park et al., 2017; Parodi et al., 2017; Armstrong and Stevens, 2018; Usman et al., 2018; Cao and Liu, 2020; Fdez-Gubieda et al., 2020). Among these agents, bacteria are widely present in the ecosystem and the ease with which they can be cultured, genetically engineered, and modified makes them an attractive choice (Chien et al., 2017; Zhou et al., 2018). Moreover, several bacteria are known to colonize the host tissue effectively, which can be exploited to invade difficult places for drug penetration including the TME, but precise control is an issue (Min et al., 2008; Leschner et al., 2009; Ganai et al., 2011; Song et al., 2018). One of the bacterial groups referred to as magnetotactic bacteria (MTB) are known to naturally biomineralize magnetite crystals internally and use them as a magnetic compass for navigation, utilizing the geomagnetic field (Martel et al., 2006; Faivre and Schüler, 2008; Macwan et al., 2014; Fdez-Gubieda et al., 2020). Although some species of MTB might be difficult to grow and maintain in laboratory conditions (Lefèvre Christopher and Bazylinski Dennis, 2013), the ease of culturing and using certain species for various experiments has been documented (Matsunaga et al., 1991; Schleifer et al., 1991; Liu et al., 2010; Zhang et al., 2011; Ali et al., 2017; Fdez-Gubieda et al., 2020). The motility of MTB cells can be precisely controlled by locally generating a magnetic field that is few times stronger than the geomagnetic field. In this study, we show the directional control of MTB using ∼3-mm-sized solenoid coils forming a network of tracks and the ability of these MTB cells to interact with the mammalian cells using an *in vitro* monolayer of Chinese hamster ovary (CHO) cells. The specific interactions between the species *Magnetospirillum magneticum* (AMB-1) and CHO cells are investigated using a molecular dynamics (MD) approach, where interactions between two prominent bacterial surface proteins (bacterial cell surface protein, MSP-1 and flagellar protein, flagellin) and CHO cell cytoplasmic and extracellular proteins (BSG, B2M, SDC1, AIMP1, and HTR1B) are studied. Based on the experimental and MD results, it is anticipated that AMB-1 cells can isolate the mammalian cells and, by extension, tumor cells from a group of healthy cells. It is important to note that the invasion of CHO cells by AMB-1 is proposed as an invasion of the microenvironment of CHO cells by the AMB-1 cells and not the actual internalization of the AMB-1 cells.

## Materials and Methods

### Cell Culture

AMB-1 and CHO cells were purchased from the American Type Culture Collection (ATCC). AMB-1 cells were cultured in revised magnetic spirillum growth medium (MSGM) and maintained at 30°C following ATCC protocol. The MSGM was prepared based on ATCC recommendations and the media pH was maintained at 6.75 using 0.1 M sodium hydroxide. CHO cells were cultured in F-12K medium supplemented with 10% fetal bovine serum and 1% antibiotics and maintained in 5% CO_2_ and at 37°C. When the cells were in exponential growth phase, they were collected using trypsin and used for further experiments.

### Characterization of AMB-1 Bacteria

To verify the magnetotaxis response from AMB-1 cells, we used a permanent magnet. After the fourth day of inoculation of AMB-1 cells, we observed a small gray sediment at the bottom of the test tube. Magnet was placed near the bottom of the tube and we observed the response of AMB-1 cells in stimuli to the magnet’s movement. For visualizing the morphology, AMB-1 cells were stained using crystal violet. A 100-μl sample of the culture was used to make a smear on the glass slide and allowed to air dry inside the laminar flow hood. The smear was stained with a drop of crystal violet solution for 1 min and washed with DI water. The morphology was observed under a light and inverted microscope (ZEISS Primovert). Hanging drop assay was used to investigate the motility of AMB-1 cells. A tiny drop of AMB-1 cells was placed on the cover slip and then a small amount of petroleum jelly was used on the sides of the cover slip and sealed with a concave-shaped slide from top. The concave glass slide was turned around and the drop was found hanging on the square-shaped cover slip. The motility of AMB-1 was then observed using a light microscope at 100 × magnification using immersion oil. The growth curve for AMB-1 was obtained from the optical density (OD) measurements at 600 nm wavelength.

### Invasion Assay

AMB-1 cells were incubated with CHO cells in a 2:1 ratio for 2, 8, and 24 h separately on a 24-well plate. Appropriate controls for AMB-1 and CHO cells were also included in the experiment. The experiment was repeated for a total of three times to account for biological variations. The samples were taken from each well when the incubation time is completed to perform quantitative analysis. For the quantitative analysis of the co-incubation of CHO cells with AMB-1, the number of viable cells for both CHO and AMB-1 was counted before and after each incubation period using lactate dehydrogenase assay. To 10 μl of the culture sample, 10 μl of 0.4% trypan blue staining solution is added and the mixture is incubated for ∼3 min. Trypan blue will not pass through the membrane of viable cells while dead cells have no intact membrane and hence pick up trypan blue and get stained to blue (Strober, 2015). Ten microliters of this mixture is placed on a hemocytometer, and viable cells (unstained) are counted. The co-incubated and control samples from each incubation period were also prepared for scanning electron microscopy (SEM) and energy-dispersive X-ray spectroscopy (EDAX) analysis.

### Magnetic Field Generation

The magnetic field was generated by three custom-built solenoid coils (diameter ∼ 3 mm and length ∼ 5 mm) using 32 AWG (American Wire Gauge) conductor and were placed vertically with a distance of 7 mm between each other in a 35 mm × 10 mm petri dish. The magnetic field generated by the coils was controlled using a field programmable gate array (FPGA) chip on the Altera DE-II board, which supplied a current of ∼1 A. The FPGA was also utilized to switch the magnetic poles on the coils by changing the direction of the flow of current. This switching of current and hence the magnetic field was used to establish the magnetic path for controlling the direction of the AMB-1 cells. For directional control of AMB-1 and co-incubated AMB-1/CHO cells, a drop of the sample is placed in the center of petri dish and magnetic field is applied while continuously monitoring the region under an inverted microscope.

### Scanning Electron Microscopy

Eight hundred microliters of the sample from AMB-1 control, CHO control, and AMB-1 co-incubated with CHO for 24 h was collected in an Eppendorf tube. The samples were centrifuged at 3,000 rpm for 3 min to remove the supernatant. The pellet was washed with phosphate buffered saline (PBS) once and fixed in 4% glutaraldehyde at 4°C for 4 h. The sample was washed with PBS one more time and this time fixed in 2% glutaraldehyde at 4°C for 1 h. After fixation, samples were rinsed in PBS three times and then dehydrated using serial incubations in increasingly concentrated ethanol solutions (20%, 50%, 75%, and 100%) for 10 min and centrifuged at 3,000 rpm for 3 min between subsequent incubation. Finally, the sample is infiltrated with tert-butanol to freeze dry the sample (Baskin et al., 2014). Before imaging, the sample was mounted on SEM stub and coated with gold nanoparticles for enhanced imaging. A Phenom ProX desktop SEM integrated with EDAX was employed to observe the morphology of the sample. After imaging, elemental analyses were performed using the same Phenom ProX system equipped with thermionic source (CeB_6_) operating at an accelerating voltage of 10 kV (imaging mode) and a backscattered electron detector.

### Transmission Electron Microscopy

For TEM imaging, samples were prepared based on SEM procedure described in the previous section until the infiltration of samples with tert-butanol. The samples were then directly mounted on the TEM carbon-coated copper grids. The samples were allowed to dry and then they were observed under the TEM (Analytical Bio/Soft TEM 120 kV, JEOL 1400) to determine the presence and size of magnetosomes.

### MD Simulations

For simulating the interactions between bacterial and mammalian cell surface proteins, visual molecular dynamics (VMD) and nanoscale molecular dynamics (NAMD) were used (Phillips et al., 2020). The protein sequences for CHO cells for both extracellular and cytoplasmic proteins were obtained from UniProt and used to generate the PDB files using I-TASSER (Yang et al., 2015; UniProt Consortium., 2021). Later, the protein structure files (PSFs) were generated by utilizing the PDB and topology files based on the CHARMM (Chemistry at HARvard Macromolecular Mechanics) force field parameters. All atom simulations were carried out between the AMB-1 cell surface proteins (MSP-1 and flagellin) and each of the three extracellular CHO proteins (B2M, SDC1, and AIMP1) and plasma membrane proteins (ATP7A, BSG, CD44, CDH2, GJC1, HTR1B, and LAMP1). Supplementary Table 2 shows the number of atoms for each of the simulated systems including water and ions. The water molecules were modeled using the TIP3 (Transferable Intermolecular Potential, point 3) and a neutralizing concentration of NaCl was used to polarize the water molecules. The simulations were carried out partly on the XSEDE (Extreme Science and Engineering Discovery Environment) supercomputing facility that provided access to 54 CPU pairs with 28 cores per CPU (total 1,512 cores) supercomputer cluster and on Intel core i5 (16 core) machines equipped with GPU acceleration (Towns et al., 2014). The temperature was set to 300 K and maintained by Langevin Thermostat and the pressure was maintained at 1 atm through a Nose-Hoover Langevin-piston barostat with a period of 100 ps and a decay rate of 50 ps. An integrated time step of 2 fs was maintained for all simulations. A cutoff of 10–12 Å was used for short-range forces and particle mesh Ewald algorithm was used for calculating long-range forces. A 5,000-step energy minimization was performed first on all systems to reach a minimum for the potential energy followed by an equilibration of 500,000 steps (1 ns). Root mean square deviation (RMSD) and NAMD energy extensions were used from VMD to determine the interaction energies between the different protein molecules. Additionally, the TimeLine tool from VMD was utilized to analyze the secondary structure of the proteins as they interacted with each other. Interaction energies, optimum cutoff distances, and distances between the center of masses of individual proteins were quantified using TCL scripts.

## Results and Discussion

### Characterization of AMB-1 Bacteria

AMB-1 culture appeared as a gray sediment at the bottom of the test tube after 4 days of inoculation. AMB-1 cells swirled toward the magnet as soon as a small magnet is brought closer to the tube. As the magnet is moved, the AMB-1 closely followed the magnet through magnetotaxis. The movement of AMB-1 in response to magnet is captured in Supplementary Video 1. The morphological analysis of AMB-1 with crystal violet revealed the size and spiral shape of the bacteria (Supplementary Figures 1A,B). Further information about the bacterial structure was obtained from the TEM, SEM, and EDAX analysis. Similarly, when we observed the hanging drop under the light microscope, we could see the erratic movement of AMB-1 cells confirming the motility of the bacteria (Supplementary Video 2). The growth curve of AMB-1 over time is shown in Supplementary Figure 1C.

### Directional Control of Co-incubated AMB-1 CHO Cells

**FIGURE 1:**
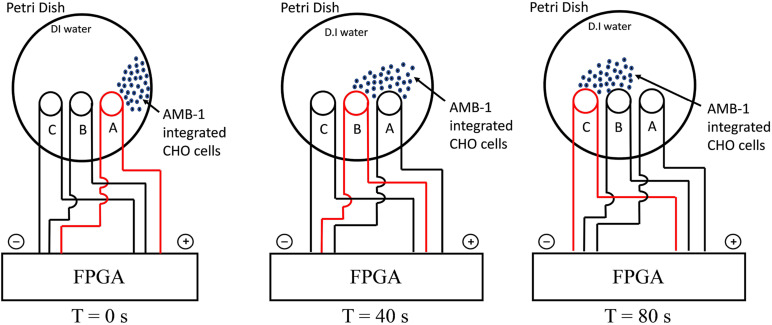
Schematic illustrating the experimental setup for directional control displaying the movement of AMB-1-integrated CHO cells from coil A to coil C. The charged coil is showed in red, and the AMB-1-integrated CHO cells are displayed as blue dots.

**FIGURE 2:**
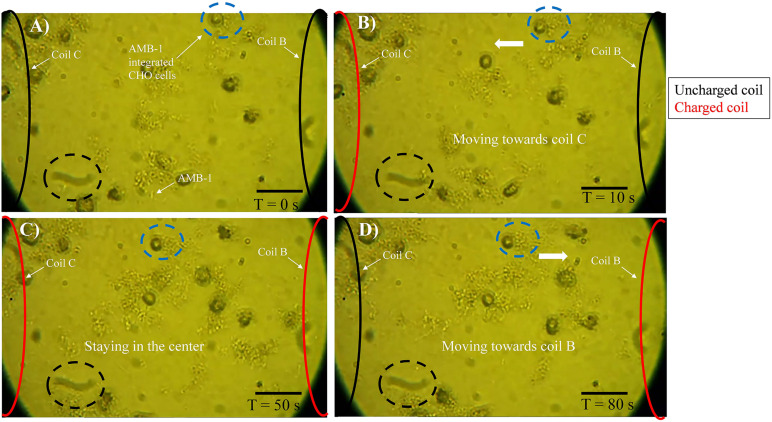
Motility of AMB-1-integrated CHO cells with time. **(A)** At *T* = 0 s, AMB-1-integrated CHO cells are moving randomly. **(B)** At *T* = 10 s, coil C on the left is charged and an AMB-1-integrated CHO cell, highlighted by a blue dotted circle, starts to navigate toward the charged coil C. **(C)** At *T* = 50 s, coils B (right) and C (left) are charged and the AMB-1-integrated CHO cell in the blue dotted circle stays in the center. **(D)** At *T* = 80 s, only coil C (left) is discharged and the AMB-1-integrated CHO cells can be seen moving toward coil B to the right. A stationary black dotted circle is added at the bottom of every frame to serve as a reference point. Reversal was achieved in the same manner (scale bar = 50 μm). Supplementary Videos 3, 4 show this controlled navigation of AMB-1-integrated CHO and AMB-1 cells, respectively.

The experimental setup used in the study is shown in Supplementary Figure 2 and the schematic of the entire methodology is illustrated in Figure 1. The FPGA was programmed using Verilog hardware description language and used for controlling the coils in the petri dish individually. As the coil is supplied by current from FPGA, it creates magnetic poles, and the AMB-1 cells move toward the coil *via* magnetotaxis. In Figure 1, the magnetic field lines are diverted from one end of the petri dish to another by choosing which coil is being supplied by the current through the FPGA. As the magnetic pole, represented by the top of the individual coil, moves, the AMB-1 cells move toward the pole almost instantly. Figure 2 shows the images acquired under the phase contrast (inverted) microscope at 40 × magnification during the invasion assay. At time *T* = 0 s, AMB-1 cells exhibit Brownian motion and interact with the neighboring CHO cells. When the coil B is charged at *T* = 10 s, the CHO cells integrated with AMB-1 bacteria move toward coil B (marked with a dashed circle). The CHO cells continue to move as the magnetic pole is now shifted toward the other end of the petri dish. The direction of the cells is reversed by switching the order of the coils in the other direction. Supplementary Videos 3, 4 capture in detail the directional control of AMB-1-integrated CHO cells and AMB-1, respectively.

### Characterization of AMB-1-Integrated CHO Cells

**FIGURE 3:**
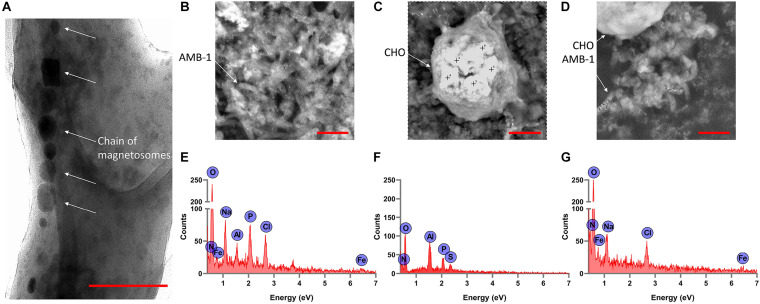
Characterization of individual AMB-1 cells and AMB-1/CHO cell interactions using TEM and SEM. **(A)** TEM analysis of AMB-1 shows the presence of the chain of magnetosomes, as pointed out by the white arrows (scale bar = 0.1 μm for A); SEM images of **(B)** AMB-1 control, **(C)** CHO control, and **(D)** AMB-1 with CHO cells [scale bar = 2 μm for panels **(B,D)** and 3 μm for panel **(C)**] (the “+” sign on the SEM images shows the spots at which EDAX spot analysis is performed); EDAX analysis of **(E)** AMB-1 control, **(F)** CHO control, and **(G)** AMB-1 with CHO cells (signal from carbon has been removed to make the low-intensity peaks more visible).

From the TEM analysis of AMB-1 cells, we can clearly visualize the visualize the presence and size of magnetosomes and magnetite crystals (Figure 3A). The fixed AMB-1-integrated CHO cells were further analyzed using SEM. Figures 3B–D show the SEM images of the fixed AMB-1, CHO control, and AMB-1-integrated CHO cells, respectively. CHO cells appeared as white spheres with a diameter of ∼14–15 μm while AMB-1 was observed to be having a spiral morphology and having cellular lengths ranging from 6 to 9 μm and diameters from 0.5 to 0.75 μm comparable to the known morphology. With the help of EDAX, the presence of magnetosomes was also confirmed with signature peaks of iron and a chain of such crystals aligned along the long axis of the bacterial cell. Furthermore, Figure 3D shows the presence of several AMB-1 bacteria around the cell membrane of CHO cells. This shows that AMB-1 cells not only remain alive but also retain magnetotaxis near the cell membrane of the CHO cells. Several spots were chosen on the SEM images and a thorough EDAX analysis was performed to characterize the elements inside the CHO and AMB-1 cells. The results of the EDAX analysis for AMB-1 cells, CHO cells, and AMB-1-integrated CHO cells are displayed in Figures 3E–G, respectively. EDAX analysis (Table 1) revealed the presence of iron in the samples of AMB-1-integrated CHO cells. The presence of these iron complexes is indicative of the magnetosomes from AMB-1 as such iron signatures were not found in CHO control samples. Additionally, compared to CHO control, AMB-1 and AMB-1-integrated CHO show increased oxygen peaks, and this increase in oxygen peak also indicates the presence of magnetosomes (magnetite crystals, Fe_3_O_4_). These results provide important insights into the successful interactions and thus association of CHO cells by AMB-1.

**TABLE 1:** Results of the EDAX analysis revealed the elements and their concentration in CHO cells, AMB-1, and AMB-1-integrated CHO cells.

Element			AMB-1 control		CHO control		AMB-1-integrated CHO	
Atomic number	Symbol	Name	Atomic conc.	Weight conc.	Atomic conc.	Weight conc.	Atomic conc.	Weight conc.
6	C	Carbon	55.71	46.8	68.9	62.21	69.48	62.53
8	O	Oxygen	25.88	28.96	14.19	17.07	15.24	18.27
7	N	Nitrogen	14.29	14	14.3	15.05	13.56	14.23
26	Fe	Iron	0.85	3.31	–	–	0.66	2.78

The viability of both AMB-1 and CHO cells post co-incubation and statistical analysis of data are tabulated in Supplementary Table 1. Initially, AMB-1 cells decreased in population after 2 h post co-incubation with CHO cells, and this decreasing trend was observed at the 8-h time point as well. After 24 h, a total of 51% of AMB-1 cells was found viable. Similar to AMB-1 cells, the number of viable CHO cells was decreasing at each time point and an overall 67% of cells were found viable at the final 24-h time point. Interestingly, the number of viable AMB-1 cells at the 24-h time point was 26% higher than the AMB-1 control, and this observation was opposite for CHO cells, which is 62% lower than the control group. Although no statistical significance was found in the viability values among different incubation periods (Supplementary Figure 3), descriptive statistical analysis resulted in a larger deviation than expected among three experimental trials. This may be due to both the biological variations arising from the cells and experimental variations that occurred in sampling for cell counts at various time points. AMB-1 cells grow rapidly and are observed to survive in an environment other than their own (Fdez-Gubieda et al., 2020). Overall, we expect that the viability results could help us in understanding the possibility of interactions between AMB-1 and CHO cells. Furthermore, the positive results of invasion assay can be advantageous for controlled tumor targeting by targeting the tumor cells by means of bacterial magnetotaxis. In the future, atomic force microscopy can be used to study the interaction of AMB-1/CHO cells at the molecular level.

### MD Simulation

**FIGURE 4:**
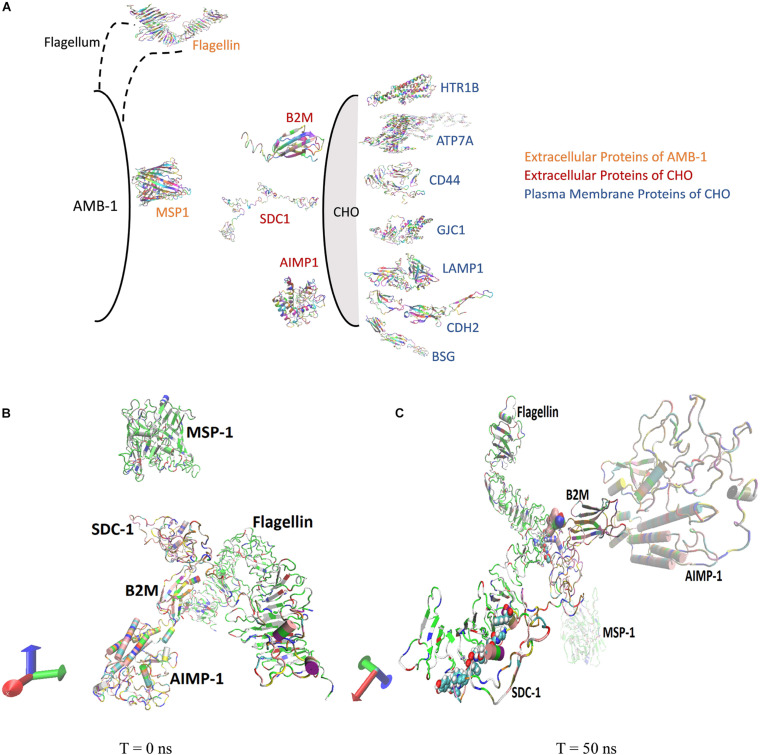
**(A)** AMB-1 and CHO surface proteins used for performing molecular dynamics simulation. **(B)** Spatial location of proteins at time **(B)** *T* = 0 ns and **(C)** *T* = 50 ns, respectively.

Molecular dynamics simulations are conducted to understand the interactions between the AMB-1 and CHO cells as seen from the experiments. Existing literature surveys confirm the presence of MSP1 and flagellin proteins (comprising ∼80%) on the outer membrane of AMB-1 as surface proteins. Flagellin is a flagellum protein and has shown to have high-affinity interactions with the cell receptors whereas MSP-1 is responsible for maintaining proper cell organization and structure (Steiner, 2007; Ando et al., 2014; Ozden et al., 2017; Wang et al., 2020). For selection of the CHO proteins, out of the thousands of them, 14 proteins were pressed down and further categorized as extracellular and plasma membrane proteins based on the location of the proteins and available data reviewed by UniProt (UniProt Consortium., 2021). The conformational and structural changes in both extracellular and plasma membrane proteins at the interface of CHO and AMB-1 surface gave crucial insights into the way the bacterial and CHO cells may have interacted. Furthermore, the quantification of the stability and energy aspects between these cell surface proteins also sheds light on the reasons as to why AMB-1 cells were able to associate with the CHO cells, making them magnetic. Stability of the interface is known through the RMSD, hydrogen bonds, center of mass, and secondary structure analysis for the proteins. Figure 4A is a diagrammatic representation of the extracellular and plasma membrane proteins of CHO cells and AMB-1. Figures 4B,C show the beginning (0 ns) and end of run (50 ns) screen captures of the simulation interaction for extracellular proteins displaying the change in the trajectories of the proteins. Similar screenshots for CHO cells plasma membrane proteins for time points *T* = 0 ns and *T* = 50 ns are shown in Supplementary Figure 4.

### Interaction of AMB-1 Surface Proteins With CHO Extracellular Protein

**FIGURE 5:**
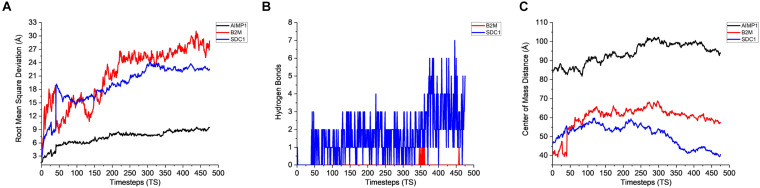
Stability analysis for the interactions between CHO extracellular proteins and AMB-1 surface proteins. **(A)** Root mean square deviation (RMSD), **(B)** hydrogen bonds, and **(C)** center of mass (1 TS = 105 ps).

**FIGURE 6:**
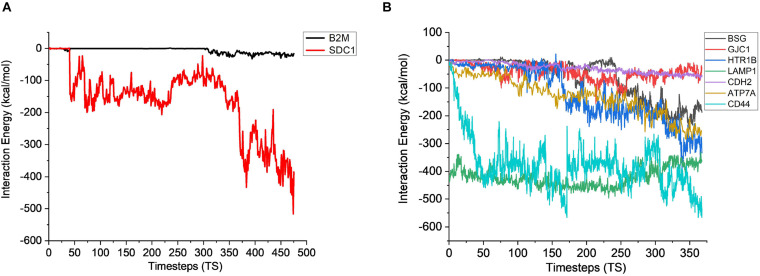
Interaction energy analysis for CHO **(A)** extracellular proteins (1 TS = 105 ps) and **(B)** plasma membrane proteins with AMB-1 bacteria (1 TS = 136 ps).

The stability analysis for extracellular and plasma proteins marks the first step in the quantification of the interface between CHO and AMB-1 cells. As shown in Figure 5A, both B2M and SDC1 CHO extracellular proteins undergo large initial deviations up to 26 and 24 Å during the first 23.62 and 31.5 ns, respectively. Each extracellular protein is responsible for different functions that together contribute toward proper functioning of the cell. Functions such as ion transport, immune response, wound repair, wound healing, regulation of cellular senescence, and inflammatory response of CHO cells thereby indicate that the invasion by AMB-1 would trigger the immune system and alarm the CHO cell about the existence of a foreign entity. B2M is believed to trigger the cellular immune response by binding antigenic peptides, presenting them to the T-cell receptors (Mohd-Padil et al., 2010); SDC1 displayed identical and protein C-terminus binding, and inflammatory response is one of the major functions of AIMP1 (Zhang et al., 1995; Quevillon et al., 1997). Furthermore, in contrast to these two extracellular proteins, AIMP1 shows minor deviation until 21 ns. Once the proteins find a favorable conformation and location on the AMB-1 protein complex, the proteins start to stabilize with minor fluctuations as they first encounter the protein flagellin on the AMB-1 complex. Based on the number of hydrogen bonds for these proteins from Figure 5B, it is evident that the number of hydrogen bonds double for SDC1. For B2M and AIMP1, it is found that the number of hydrogen bonds increases around 350 ns as it comes closer to flagellin, whereas for AIMP1, it is far away from both the AMB-1 proteins, showing no interaction. To quantify the affinity of these extracellular proteins toward AMB-1 proteins, the center of mass analysis was performed as shown in Figure 5C, which indicated that the protein moves toward each other, reducing the distance between each other and stabilizing at the spot well. The graph displays the interaction of the extracellular protein with flagellin as for B2M, and SDC1 shows that after initial interaction, it can find a favorable spot and continues to stabilize with flagellin whereas AIMP1 moves further away. Finally, protein secondary structure analysis helps conclude the stability of the proteins where the changes in the secondary structure are quantified (Supplementary Figure 5). Each helix or turn in a protein’s structure is responsible for a particular function such as domination or suppression. Secondary structure changes were not observed for other extracellular proteins (B2M) except the analysis for SDC1 demonstrates that the protein is vulnerable to lose its function of cell signaling owing to a massive conformational change resulting into an exchange between a coil and a turn (Supplementary Figures 5A,B). As per this change, the protein will be unable to report the changes in its microenvironment and in turn aiding in stabilizing the interactions. It is thus anticipated that the structure conversion of coil to extended beta and finally to turn causes the loss of cell binding and cell signaling. The total interaction energy plot provides information on the non-bonded interaction energy, which includes a sum of van der Waals and electrostatic energies. The van der Waals forces define the interactions between the molecules, being attractive or repulsive, whereas the potential energy landscape provides the information on the internal energy of the protein (Phillips et al., 2005, 2020). The energy plot for extracellular proteins from Figure 6A depicts the interactions between SDC1 and flagellin, which is the most negative and interacts the most with energy up to 500 kcal/mol compared to B2M and AIMP1. This indicates that the disabling of the signaling function in CHO extracellular proteins may have played a huge role in their control by AMB-1.

### Interaction of Surface Protein of AMB-1 With the Plasma Membrane Proteins of CHO Cells

**FIGURE 7:**
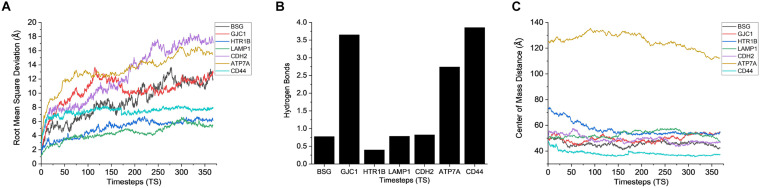
Stability analysis for the interactions between CHO plasma membrane proteins and AMB-1 surface proteins. **(A)** Root mean square deviation (RMSD), **(B)** hydrogen bonds, and **(C)** center of mass (1 TS = 136 ps).

After gaining sufficient knowledge on how the surface proteins of CHO and AMB-1 are interacting, the simulated trajectories for the interactions between CHO plasma membrane proteins (HTR1B, ATP7A, BSG, CDH2, CD44, GJC1, and LAMP1) and AMB-1 proteins were analyzed. As can be noted from Figure 7A, the plasma membrane proteins interacted more with MSP1 in comparison to flagellin. The proteins on their RMSD analysis start to stabilize after multiple fluctuations and deviations at different nanoseconds as they find a favorable spot. Each protein stabilizes to a favorable spot at different time steps, e.g., HTR1B, LAMP1, and CDH2 seem to start stabilizing around 29 ns whereas GJC1 and CD44 stabilize around 23 ns. The protein then interacts with the surface proteins of AMB-1, and water and the interactions are visible with the increase in the number of hydrogen bonds (Figure 7B). The interaction of GJC1, ATP7A, and CD44 with MSP1 can be seen with the highest increase in hydrogen bonds, which implies that these proteins are interacting with more likeability and compatibility. With reference to Figure 7C, the center of mass between the interacting proteins reduces for almost all the CHO proteins, thereby moving toward each other. HTR1B (Serotonin) is a multifunctional bioamine that binds to and activates multiple receptor subtypes with diverse physiological consequences in the central nervous system and peripheral tissues (Obberghen-Schilling et al., 1991) whereas BSG, also known as CD147, has a function of signaling receptor activity (Pushkarsky et al., 2001). While there were no changes in the secondary structure for BSG, the secondary structure analysis for HTR1B shows that the conversion of pi helix to alpha helix leads to loss of that function (Supplementary Figures 5C,D). Since the cell loses its structure and, in turn, its function, it fails to notify about the change in its microenvironment during a potential invasion by the AMB-1 cells. CHO cell is unaware about this invasion as it has lost its signaling receptors and hence continue to multiply even in the presence of AMB-1, which is also seen from the growth curve. The average interaction energy for plasma membrane proteins shows that the interactions are stable with MSP1 with high interaction energies, e.g., ATP7A continues to interact with increasing negative energy after 10 ns, whereas for other proteins such as HTR1B, GJC1 and BSG stabilize and interact around 40 ns as shown in Figure 6B. With the VMD simulation results, it is observed that the extracellular proteins interact with flagellin whereas plasma membrane proteins interact more with MSP1. This behavior leads to the possibility that flagellum is lost after the interaction with extracellular protein, which then allows the membrane-specific MSP1 protein to interact more with plasma membrane proteins.

The final step toward the analysis is to know whether there is a change in the secondary structure of the protein. The secondary structure of any proteins holds information about the function/role of that protein and how its change can lead to loss of the function and in turn its survival in that environment. The timeline analysis for two extracellular and two plasma membrane proteins is shown in Supplementary Figure 5. The CHO extracellular protein, SDC1, is responsible for cell signaling, and from the plot, structural change from turn to coil is observed. This change states that the conformational change is compromising its function to indicate the CHO cell that it has been manipulated. With the failure of proper cell signaling, CHO cells fail to put up a defense shield, being completely unaware of the foreign body, and are under the assumption of being in a favorable environment to multiply, thereby surviving in a new media. Similarly, the CHO plasma membrane protein that performs the function of nociception and immunological responses is down. The loss of the function leads to the failure of reporting to the cell and hence the survival of the cell. The promising and reproducible experimental results show that the directional control is achieved for the AMB-1. Further exploration of VMD simulations confirms the behavior of the species during the experimental procedure. With the information on the protein structures combined with cell signaling function, further research can be done toward various bioengineering applications. The biocompatibility of the bacteria can be used as a vector for controlled and regulated drug delivery. Also, meticulous analysis using computational biophysics of CHO and AMB-1 plasma membrane proteins for their reactive or binding sites will be helpful in knowing function accountability.

## Conclusion

Based on the controlled navigation capabilities of AMB-1, a magnetic invasive assay to target a group of mammalian cells containing both healthy and tumor cells is envisaged. Interactions at the interface of MSP-1 and flagellin proteins in AMB-1 indicated that there is an interesting difference between the way AMB-1 cells interacted with CHO extracellular and plasma membrane proteins, respectively. It is anticipated that the mammalian cell surface proteins, which are predominantly responsible for cell signaling, are the primary targets of the AMB-1 cells that are dissuaded by flagellin of AMB-1. On similar lines, the plasma membrane proteins, whose primary function is to maintain the mammalian cell structure and function, are the targets of the AMB-1 cell surface protein, MSP-1. With the obtained results, future goals and challenges would be to use AMB-1 with a group of mutated CHO cells and observe them for isolation from normal healthy cells.

## Data Availability Statement

The original contributions presented in the study are included in the article/Supplementary Material, further inquiries can be directed to the corresponding author/s.

## Author Contributions

IM conceptualized and designed the study. MX designed hardware interface and programmed FPGA. MX and HD executed the experimental part for conceptual verification of magnetotaxis. HD modeled and performed VMD and NAMD simulations. HD and IM performed the data analysis on the simulated trajectories. MH and IM performed SEM imaging. MX, IM, and MH performed EDAX analysis. MH and SS contributed toward the wet lab experiments. MX, HD, and MH drafted the manuscript. All authors contributed to revise the manuscript and approved the submitted version with proofreading performed by IM.

## Conflict of Interest

The authors declare that the research was conducted in the absence of any commercial or financial relationships that could be construed as a potential conflict of interest.
