# Supplementary material for: Towards Tumor Targeting via Invasive Assay Using Magnetospirillum magneticum
Source: Front Microbiol. 2021 Jul 22;12:697132. doi: 10.3389/fmicb.2021.697132 (PMC8341810; doi:10.3389/fmicb.2021.697132)
Supplement: Supplementary file 1 [file Data_Sheet_1.docx]

**Towards tumor targeting via invasive assay using *Magnetospirillummagneticum***

Marvin Xavierselvan^1^, Heena R. Divecha^2^, Mamta Hajra^2^, Sushila Silwal^2^, Isaac Macwan^3*^

^1^Department of Biomedical Engineering, Tufts University, Medford, MA, USA

^2^ Department of Biomedical Engineering, University of Bridgeport, Bridgeport, CT, USA

^3^Department of Electrical and Biomedical Engineering, Fairfield University, Fairfield, CT, USA

***Correspondence:**[imacwan@fairfield.edu](mailto:imacwan@fairfield.edu)

**Supplementary Figures:**

**Figure S1:** AMB-1 bacteria imaged at magnification A) 100x objective (light microscope) and B) 40x objective (phase contrast inverted microscope). C) Growth curve of AMB-1 bacteria over time.

**Figure S2** showing the experimental setup used in the study. In performing the actual experiment, FPGA replaces the power supply for AMB-1 directional control.


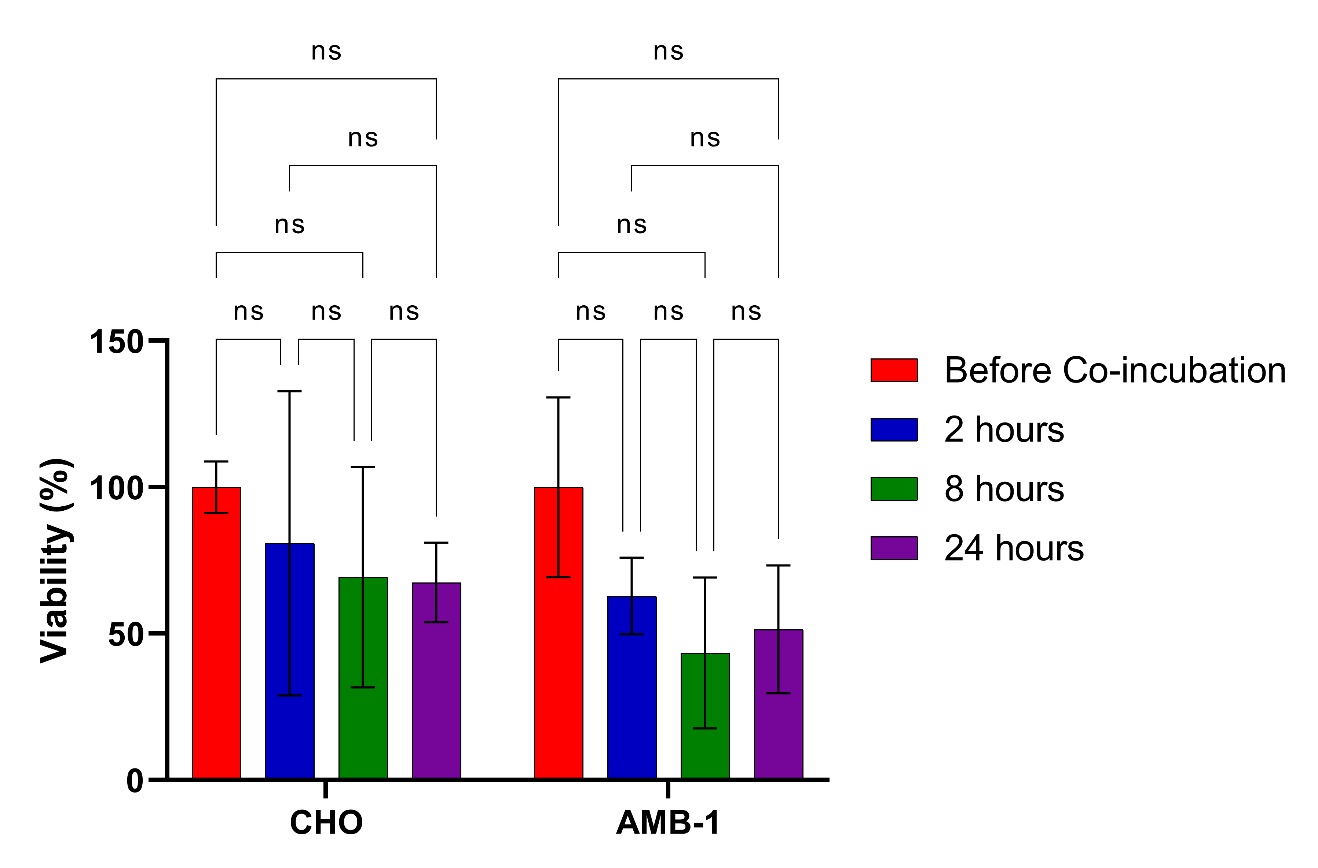


**Figure S3:** Percent viability of CHO and AMB-1 cells before and after co-incubation. Normalized to before co-incubation viability. Two-way ANOVA with Tukey’s multiple comparisons test was performed to test out statistical significance among the viability of different incubation periods. No significance (ns) was observed among different incubation periods.


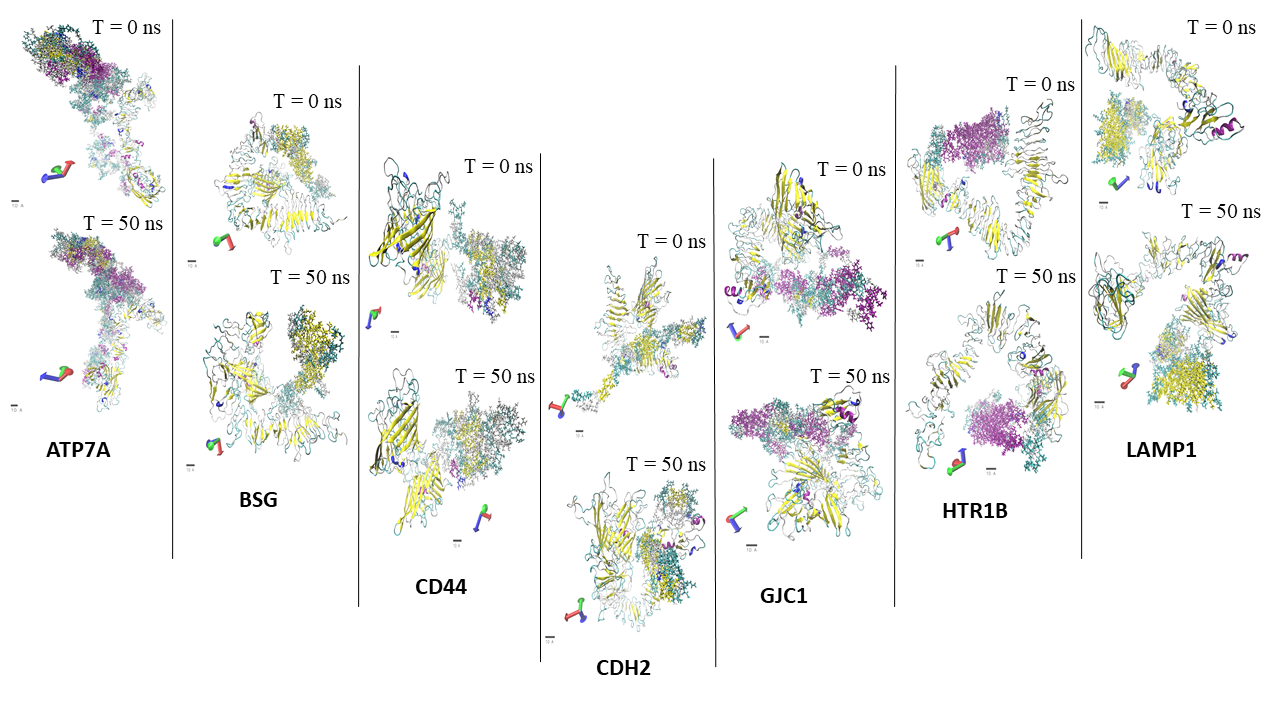


**Figure S4:** Visual Molecular Dynamics simulation results for AMB1 and CHO plasma membrane proteins at time points T=0ns and T=50ns.

**Figure S5:** Timeline Analysis of CHO extracellular proteins, A) B2M & B) SDC1, and plasma membrane proteins, C) BSG & D) HTR1B, showing the secondary structural changes in protein structure.

**Supplementary Tables:**

**Table S1:** Viability of the AMB-1 and CHO cells before and after co-incubation in terms of number of cells along with statistical analysis.The viability of controls for AMB-1 and CHO cells were evaluated 24-hour post incubation period.

| 1^st^ Trial | Before co-incubation | | | 2 hours | | 8 hours | | 24 hours | | | Control | |
| --- | --- | --- | --- | --- | --- | --- | --- | --- | --- | --- | --- | --- |
| AMB-1 | 121,104 | | | 37,833.33 | | 12,800 | | 10,666.66 | | | 41,300 | |
| CHO | 85,000 | | | 178,000 | | 138,333.33 | | 71,900 | | | 153,000 | |
|  | | | | | | | | | | | | |
| 2^nd^ Trial | Before co-incubation | | | 2 hours | | 8 hours | | 24 hours | | | Control | |
| AMB-1 | 69,003 | | | 68,400 | | 73,000 | | 68,900 | | | 21,300 | |
| CHO | 91,300 | | | 34,166.66 | | 41,333.33 | | 39,800 | | | 213,000 | |
|  | | | | | | | | | | | | |
| 3^rd^ Trial | Before co-incubation | | | 2 hours | | 8 hours | | 24 hours | | | Control | |
| AMB-1 | 40,640.63 | | | 38,733.33 | | 14,383.33 | | 39,233.33 | | | 25,000 | |
| CHO | 113,000 | | | 21,850 | | 20,866 | | 83,466.66 | | | 196,000 | |
|  | | | | | | | | | | | | |
| Percent viability | | Before co-incubation | | | 2 hours | | 8 hours | | | 24 hours | | |
|  | | Mean | Std. Error | | Mean | Std. Error | Mean | | Std. Error | Mean | | Std. Error |
| AMB-1 | | 100 | 43.32327 | | 62.82465 | 23.25659 | 43.41682 | | 28.98479 | 51.48453 | | 26.95356 |
| CHO | | 100 | 12.43701 | | 80.89066 | 52.46189 | 69.31674 | | 38.06076 | 67.46146 | | 14.78704 |

**Table S2**: Number of atoms of the simulated systems including water and ions.

| CHO Protein | Location | Total number of atoms  = CHO protein + MSP1 + Flagellin + water + ions |
| --- | --- | --- |
| B2M | Extracellular protein | 433841 |
| SDC1 | Extracellular protein |  |
| AIMP1 | Extracellular protein |  |
|  | | |
| HTRP1 | Plasma membrane protein | 179514 |
| ATP7A | Plasma membrane protein | 644296 |
| BSG | Plasma membrane protein | 155394 |
| CDH2 | Plasma membrane protein | 201726 |
| CD44 | Plasma membrane protein | 146806 |
| GJC1 | Plasma membrane protein | 175146 |
| LAMP1 | Plasma membrane protein | 171675 |

**Supplementary Video:**

**Video S1:** Video demonstrating the verification of magnetotaxis of AMB-1. AMB-1 swirls along the magnet through magnetotaxis.

**Video S2:**Video demonstrates the motility of AMB-1 using hanging drop assay.

**Video S3:** Video capturing the directional control of AMB-1 integrated CHO cells by changing the direction of magnetic field. Timeline legends for video S3. {Video starts with all coils ‘OFF’ and the field of view is between coil B (right) and C (left). At T = 0 seconds, we observe the random motion of AMB-1 integrated CHO cells. At T = 10 seconds, coil C (left) is turned ‘ON’ and we observe that AMB-1 integrated CHO starts to move left towards coil C. At T = 50 seconds, coil B (right) is also turned ‘ON’ and we observe AMB-1 integrated CHO cells staying in the center. At T = 80 seconds, coil C (left) has been turned ‘OFF’ and we observe AMB-1 integrated CHO cells moving right towards coil B.}

**Video S4:** Video showing the response of AMB-1 cells to the changes in the magnetic field in the surrounding environment. Timeline legend for video S4. {Video starts with coil A (top) being ‘ON’ and we can observe the upward motion of AMB-1 towards coil A. At T = 54 seconds, the video is now focused between coils B (bottom) and C (top) with coil B turned ‘ON’ and AMB-1 moving downwards towards coil B. At T = 59 seconds, coil C (top) is also turned ‘ON’ and AMB-1 is seen to move towards the center of the frame. At T = 1:12 (72 seconds), coil B (bottom) is turned ‘OFF’ and AMB-1 starts moving upwards towards coil C.}

**FPGA Code:**

Verilog is used to program the cyclone 2 FPGA for charging the vertical coils to produce the local magnetic field for the directional control of AMB-1 cells.

`timescale 1ns / 1ps

module MGF (clk, LEDR,GPIO_1);

input clk;

output [5:0] LEDR;

output [15:0] GPIO_1;

reg [5:0]count;

clock_divide_module U1(.clk_50Mhz(clk), .clk_1Hz(clock) );

reg a,b,c,d;

//assign

assign LEDR[0] = a;

assign LEDR[1] = b;

assign LEDR[2] = c;

assign LEDR[3] = d;

assign LEDR[4] = e;

assign LEDR[5] = f;

assign GPIO_1[0] = a;

assign GPIO_1[1] = b;

assign GPIO_1[2] = c;

assign GPIO_1[3] = d;

assign GPIO_1[4] = e;

assign GPIO_1[5] = f;

always @ (posedge clock)

begin

count <= count +1;

begin

if (count == 0)

begin

a=1; c=0; e=0;

b=0; d=0; f=0;

end

else if (count == 10)

begin

a=0; c=0; e=0;

b=1; d=0; f=0;

end

else if (count == 20)

begin

a=0; b=0; e=0;

c=1; d=0; f=0;

end

else if (count == 30)

begin

a=0; b=0; e=0;

c=0; d=1; f=0;

end

else if (count == 40)

begin

a=0; b=0; e=1;

c=0; d=0; f=0;

end

else if (count == 50)

begin

a=0; b=0; e=0;

c=0; d=0; f=1;

end

else if ( count == 60)

begin

count <= 0;

end

end

end

endmodule

`timescale 1ns / 1ps

module clock_divide_module(clk_50Mhz, clk_1Hz );

input clk_50Mhz;

output clk_1Hz;

reg clk_1Mhz_int =0;

reg clk_100Khz_int =0, clk_10Khz_int =0, clk_1Khz_int =0;

reg clk_100Hz_int =0, clk_10Hz_int =0, clk_5Hz_int =0, clk_1Hz_int =0;

reg [7:0] count_1Mhz;

reg [2:0] count_10Mhz, count_100Khz, count_10Khz, count_1Khz;

reg [2:0] count_100Hz, count_10Hz, count_1Hz;

assign clk_1Hz = clk_1Hz_int;

//Divide by 50 - 1Mhz

always@(posedge clk_50Mhz) begin

if (count_1Mhz < 50)

count_1Mhz <= count_1Mhz + 8'b00000001;

else

count_1Mhz <= 0;

if (count_1Mhz < 25)

clk_1Mhz_int <= 0;

else

clk_1Mhz_int <= 1;

end

//Divide by 10 - 100Khz

always@(posedge clk_1Mhz_int) begin

if (count_100Khz != 4)

count_100Khz <= count_100Khz + 3'b001;

else begin

count_100Khz <= 0;

clk_100Khz_int = ~clk_100Khz_int;

end

end

//Divide by 10 - 10Khz

always@(posedge clk_100Khz_int) begin

if (count_10Khz != 4)

count_10Khz <= count_10Khz + 3'b001;

else begin

count_10Khz <= 0;

clk_10Khz_int = ~clk_10Khz_int;

end

end

//Divide by 10 - 1Khz

always@(posedge clk_10Khz_int) begin

if (count_1Khz != 4)

count_1Khz <= count_1Khz + 3'b001;

else begin

count_1Khz <= 0;

clk_1Khz_int = ~clk_1Khz_int;

end

end

//Divide by 10 - 100Hz

always@(posedge clk_1Khz_int) begin

if (count_100Hz != 4)

count_100Hz <= count_100Hz + 3'b001;

else begin

count_100Hz <= 0;

clk_100Hz_int = ~clk_100Hz_int;

end

end

//Divide by 10 - 10Hz

always@(posedge clk_100Hz_int) begin

if (count_10Hz != 4)

count_10Hz <= count_10Hz + 3'b001;

else begin

count_10Hz <= 0;

clk_10Hz_int = ~clk_10Hz_int;

end

end

//Divide by 2 - 5Hz

always@(posedge clk_10Hz_int) begin

clk_5Hz_int = ~clk_5Hz_int;

end

//Divide by 10 - 1Hz

always@(posedge clk_10Hz_int) begin

if (count_1Hz != 4)

count_1Hz <= count_1Hz + 3'b001;

else begin

count_1Hz <= 0;

clk_1Hz_int = ~clk_1Hz_int;

end

end

endmodule
